# 2D Super‐Resolution Metrology Based on Superoscillatory Light

**DOI:** 10.1002/advs.202404607

**Published:** 2024-08-05

**Authors:** Yu Wang, Eng Aik Chan, Carolina Rendón‐Barraza, Yijie Shen, Eric Plum, Jun‐Yu Ou

**Affiliations:** ^1^ Optoelectronics Research Centre & Centre for Photonic Metamaterials University of Southampton Southampton SO17 1BJ United Kingdom; ^2^ Centre for Disruptive Photonic Technologies Nanyang Technological University Singapore 637371 Singapore; ^3^ School of Physics and Astronomy University of Southampton Southampton SO17 1BJ United Kingdom; ^4^ Institute for Life Sciences University of Southampton Southampton SO17 1BJ United Kingdom

**Keywords:** machine learning, optical metrology, structured light, superoscillatory light, super‐resolution

## Abstract

Progress in the semiconductor industry relies on the development of increasingly compact devices consisting of complex geometries made from diverse materials. Precise, label‐free, and real‐time metrology is needed for the characterization and quality control of such structures in both scientific research and industry. However, optical metrology of 2D sub‐wavelength structures with nanometer resolution remains a major challenge. Here, a single‐shot and label‐free optical metrology approach that determines 2D features of nanostructures, is introduced. Accurate experimental measurements with a random statistical error of 18 nm (λ/27) are demonstrated, while simulations suggest that 6 nm (λ/81) may be possible. This is far beyond the diffraction limit that affects conventional metrology. This metrology employs neural network processing of images of the 2D nano‐objects interacting with a phase singularity of the incident topologically structured superoscillatory light. A comparison between conventional and topologically structured illuminations shows that the presence of a singularity with a giant phase gradient substantially improves the retrieval of object information in such an optical metrology. This non‐invasive nano‐metrology opens a range of application opportunities for smart manufacturing processes, quality control, and advanced materials characterization.

## Graphical abstract

A single‐shot, label‐free optical metrology technique for determining 2D features of nanostructures is presented. Using neural networks processing of images of nano‐objects interacting with the phase singularity of incident superoscillatory light, it achieves measurement accuracy of 18 nm (λ/27) experimentally and 6 nm (λ/81) potentially. This offers potential applications in smart manufacturing, quality control, and semiconductor characterization.

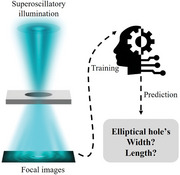

## 1 Introduction

Optical microscopy and metrology have evolved into versatile foundational tools for addressing challenges in fundamental research, manufacturing, and engineering applications, such as quality control and precision manufacturing, biostructure imaging, and medical tests.^[^ ^1^ ^]^ Advances in the semiconductor industry yield increasingly miniaturized and integrated logic and memory devices. Their increasing complexity demands rapid and accurate 2D metrology for quality control and characterization.^[^ ^2^ ^]^ Over recent decades, various metrology techniques including spectroscopic ellipsometry and reflectometry^[^ ^3^, ^4^ ^]^ as well as scanning electron microscopy (SEM) and transmission electron microscopy (TEM)^[^ ^5^ ^]^ have been extensively exploited to boost process efficiency and expedite yield ramp‐up. While SEM and TEM are employed for routine 2D/3D morphologic characterization with high resolution in the semiconductor industry, specimens subjected to high‐energy electrons may experience irreversible radiation damage.^[^ ^5^, ^6^, ^7^ ^]^ The spectroscopic inspection methods are non‐destructive and can determine the thickness or roughness of films.^[^ ^8^, ^9^ ^]^ However, these can only offer high resolution in one dimension and conventional ellipsometry and reflectometry struggle with both edge and corner areas in unit blocks due to their relatively large illuminating spot size. Fluorescent microscopies consistently achieve super‐resolution down to a few tens of nanometers and have become important tools in biomedical and semiconductor research,^[^ ^10^, ^11^, ^12^, ^13^, ^14^ ^]^ however, the necessity of introducing fluorescent labeling and follow‐up processing prevents their application in real‐time and in vivo imaging and metrology.^[^ ^15^ ^]^ Recently it has been demonstrated that a label‐free and non‐contact optical nanometrology of 1D objects with super‐resolution can be accomplished by analyzing the scattering patterns of objects with artificial intelligence.^[^ ^16^, ^17^ ^]^ Considering that the geometry of semiconductor and biological structures is usually more complex, it is desirable to develop label‐free and non‐destructive super‐resolution metrological technologies for higher‐dimensional nanoscale objects and structures.

In optical metrology, the geometry of a scatterer can be reconstructed using the Kirchhoff–Helmholtz integral equation if the intensity and phase of the scattered field are known on a closed surface surrounding the object.^[^ ^18^ ^]^ Typically, the availability of intensity information only for a limited solid angle and lack of phase information make the object reconstruction a challenging ill‐posed inverse problem.^[^ ^19^ ^]^ Very recently, machine learning, which bypasses the obstacle of the ill‐posed inverse problem by establishing a pseudo‐inverse mapping model through learning abundant training data, has been employed successfully in optical imaging and metrology, including denoising, subpixel refinement, counting and mapping, and 3D geometry recovery.^[^ ^1^, ^20^, ^21^, ^22^, ^23^, ^24^ ^]^ A fundamental aspect of machine learning in optical metrology, as a data‐driven technology, is to find the mapping relation between the actual parameters of a class of objects and the corresponding measured data. A stronger and thus more noticeable correlation between measurable data and object parameters enables the model to make more precise predictions. Recent theoretical and experimental results have revealed that topologically structured superoscillatory illumination fields yield increased sensitivity in optical dimensional and positional metrology, which suggests that they are favorable for retrieving object information from scattering patterns by machine learning.^[^ ^16^, ^25^, ^26^, ^27^ ^]^ Here, we demonstrate a single‐shot optical metrology approach that accurately retrieves 2D features of sub‐wavelength objects with an experimental random statistical error of 18 nm (λ/27) while simulations suggest that 6 nm (λ/81) may be possible. This is far beyond the Abbe–Rayleigh diffraction limit of conventional microscopy and achieved by deep learning analysis of images of objects illuminated by a phase singularity. We argue that such label‐free and non‐invasive metrology can be applied in non‐destructive testing, biomedical imaging, and characterization of semiconductor chips.

## 2 Principle and Experimental Setup

Precisely controlled multiple‐wave interference can generate a superoscillatory field with tiny and sub‐diffraction‐limited “hotspots” in free space.^[^ ^28^ ^]^ Surrounding a superoscillatory hotspot, there are large phase gradients (also known as giant local wavevectors) and large gradients of amplitude, as well as phase singularities where the phase changes from ‐π to π over distances that are a tiny fraction of the wavelength^[^ ^29^, ^30^, ^31^ ^]^ while such small optical features are absent in conventional Gaussian beams and plane waves. Recent experiments have empirically indicated that scattering patterns exhibit enhanced sensitivity to obstacles placed at positions with high amplitude gradients and/or phase gradients of the illuminating field,^[^ ^16^, ^25^, ^27^ ^]^ and this conclusion has been supported by diffraction and information theory.^[^ ^26^, ^28^ ^]^ Such enhanced sensitivity to the interaction of tiny optical features with nanoscale objects may also be anticipated for imaging of the objects, which suggests that superoscillatory illumination can improve the resolution of AI‐assisted optical microscopy and metrology.

The field distribution in the plane of the object (ellipse) may be described as *E*_Obj_ (*x*,*y*) = *E*_0_ (*x*,*y*)*g*(*x*, *y*), where incident light *E*_0_ (*x*,*y*) = *A*_0_ (*x*,*y*)exp (*i*φ_0_(*x*,*y*)) is defined by an amplitude distribution *A*_0_(*x*,*y*) and a phase distribution φ_0_(*x*,*y*), while *g*(*x*, *y*) represents the aperture function of the object. The field distribution on the camera is then given by the convolution (indicated by *) with the amplitude point spread function *PSF*(*x*, *y*) of the imaging system, *E*_Cam_ (*x*,*y*) = *E*_Obj_ (*x*,*y*)*PSF(*x*, *y*). Therefore, the intensity distribution detected by the camera is

(1) $$\begin{array}{ccc} I_{\mathrm{Cam}}\;\left(x,y\right) & = & {\left|E_{\mathrm{Cam}}\left(x,y\right)\right|}^{2}\\ & = & {\left|\left[A_{0}\left(x,y\right)\exp\left(i\varphi_{0}\left(x,y\right)\right)g\left(x,y\right)\right]*PSF\left(x,y\right)\right|}^{2} \end{array}$$

As a simple example of a small perturbation, consider a small shift of the object. For an incident plane wave, where *A*_0_ and φ_0_ reduce to constants, the small shift of the object merely results in a small shift of the image on the camera, which would be indistinguishable from a small movement of the camera. In contrast, structured illumination (i.e., non‐zero gradients of incident amplitude and/or phase) will result in more complex changes of the intensity distribution on the camera, which can be more pronounced for more rapidly varying incident light distributions, providing more object‐dependent features in detected images that machine learning can pick up on. Therefore, giant amplitude gradients and phase gradients occurring at singularities of topologically structured superoscillatory illumination of an object can be expected to contribute significantly to features of detected images that provide information about the object and may enable super‐resolution in AI‐assisted imaging and metrology.

**Figure 1:**
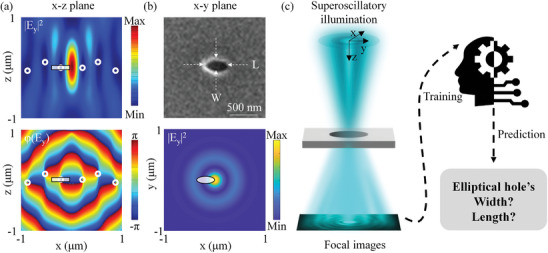
2D optical metrology via imaging with topologically structured superoscillatory light and AI analysis. a) The intensity (|*E_y_* |^2^) and phase (*φ*(*E_y_*)) profiles of the topologically structured superoscillatory light in the x‐z plane, where phase singularities are indicated by white circles. A measured elliptical aperture, represented by the rectangle, interacts with one phase singularity near the hotspot. b) SEM of a measured sub‐wavelength elliptical hole and the intensity pattern (|*E_y_* |^2^) of the superoscillatory hotspot in the x‐y plane. c) Flow diagram of the 2D optical metrology, where the superoscillatory light illuminates the measurands (different elliptical holes), and a neural network learns to retrieve the widths and lengths of elliptical apertures from their images.

Superoscillatory fields can be produced either by spatial light modulators^[^ ^16^, ^28^, ^32^ ^]^ or a static superoscillatory lens.^[^ ^33^, ^34^ ^]^ As illustrated by Figure S1 and Note S1 (Supporting Information), we employ the former approach that allows the focal hotspot to be formed at the focus of a microscope objective, offering a few hundred microns working distance, which is advantageous for scanning and measuring objects in optical microscopy systems. Superoscillatory light propagating along the z‐axis is formed from a y‐polarized Gaussian beam with wavelength λ = 488 nm via a pair of spatial light modulators, one for amplitude modulation and the other for phase. The generated superoscillatory light has a central intensity maximum, the hotspot, flanked by phase singularities that are tiny zones with high phase gradients, which are indicated by white circles in **Figure** **1**a. We utilize one phase singularity to interact with the sub‐wavelength elliptical aperture to measure its 2D size. This phase singularity exists in the energy dark zone between the hotspot and the ring‐shaped higher‐intensity halo and the hotspot is characterized by a field of view of λ and a full width at half maximum of 0.42 λ. The ground truths, the widths and lengths of elliptical holes, were established by a priori SEM measurements, and one example elliptical hole with width = 9λ/61 and length = 171λ/244 is shown in Figure 1b. We fabricated 200 sub‐wavelength elliptical holes with random sizes (i.e., widths in the 0.1 λ – 0.82 λ range and lengths in the 0.2 λ – 0.92 λ range) by focused ion beam milling of an opaque chromium film with 80 nm thickness. The sample with 200 elliptical holes is translated by a piezo stage, illuminated by superoscillatory light, and imaged by a 16‐bit camera through a transmission microscope with a 100× objective (NA = 0.9) so that the singularity of the incident superoscillatory field is focused on the object plane and the image of the object plane is collected by the camera. In experimental measurements, the alignment of elliptical apertures with the phase singularity of the illuminating superoscillatory field is vital to achieve a low statistical error with high measurement accuracy. We first find the energy dark area between the hotspot and the halo and mark the left phase singularity that will be used. Then we move the center of the elliptical aperture to align with the marked location of the phase singularity, as demonstrated in Figure 1b, Figure S2 and Note S2 (Supporting Information). To enable such optical metrology to measure unknown 2D sizes of sub‐wavelength elliptical holes via machine learning, we created a dataset including 200 single‐shot images (600*600 pixels, 7.56 µm field of view) of elliptical apertures with different sizes and randomly selected 80% as the training dataset (160 images) and 10% for validation (20 images), which were fed to the neural network for learning; subsequently, we tested the trained network's ability to retrieve the dimensions of the remaining elliptical apertures from the unseen 10% of the single‐shot images (20 images) as illustrated in Figure 1c.

A 34‐layer residual network architecture as in reference^[^ ^35^ ^]^ is employed to retrieve 2D sizes of sub‐wavelength elliptical holes. It was chosen due to its ability to efficiently learn mappings in imaging classification, object detection, and segmentation tasks.^[^ ^36^, ^37^, ^38^, ^39^, ^40^ ^]^ The initial convolutional layer consists of a 7 by 7 kernel with 64 output channels, succeeded by a 3 by 3 max‐pooling layer. It is organized into four stages, each comprising a distinct number of residual blocks: 3, 4, 6, and 3 blocks, in sequence. Within these stages, each block comprises multiple residual units, and each unit is composed of 3 convolutional layers. These layers conclude with an identity connection (shortcut connection) that bypasses one or more layers, aiding in information preservation and mitigating the vanishing gradient problem. The rectified linear unit (ReLU) serves as the activation function throughout the network, except for the final operation within each block. Features learned in the ultimate residual layer are extracted via an average pooling layer, followed by a 512 fully connected layer and a 2‐output neuron layer. The output neurons correspond to the width and length of the measurand, respectively. The network undergoes training using the Adam stochastic optimization method,^[^ ^41^ ^]^ optimizing the neural network by minimizing the mean absolute error loss function.

## 3 Results and Discussion

**Figure 2:**
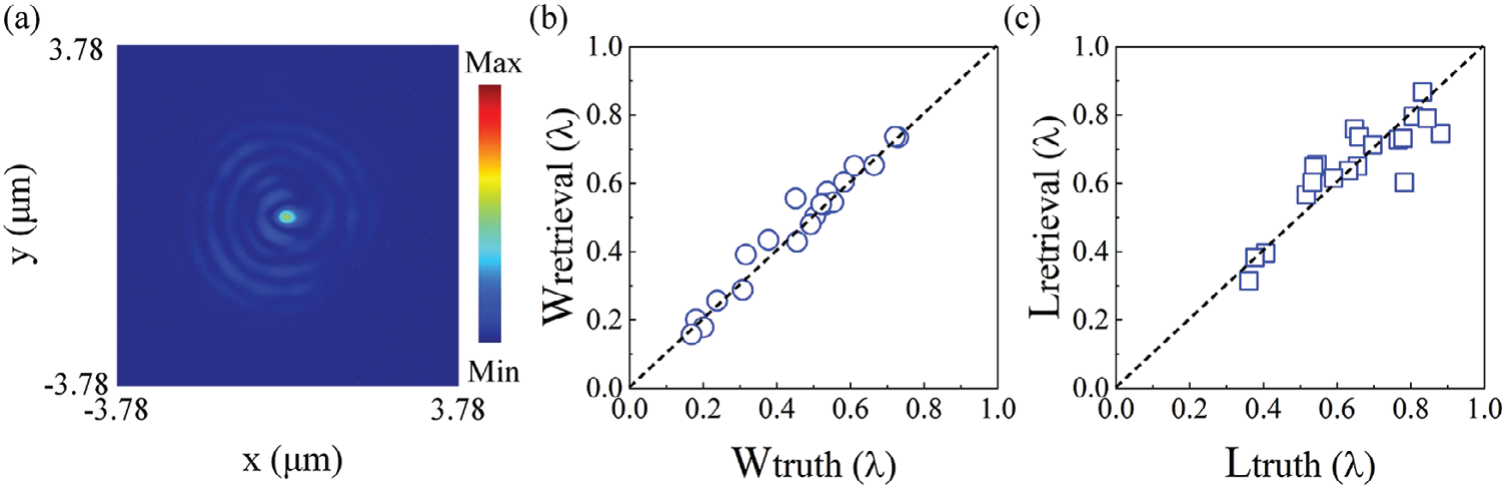
2D size retrieval of sub‐wavelength elliptical apertures based on experimental topological imaging. a) Image of an elliptical hole with width = 9λ/61 and length = 171λ/244 illuminated by the phase singularity to the left of the hotspot in the superoscillatory field. b,c) Retrieved b) widths (circles) and c) lengths (squares) of elliptical apertures versus their actual sizes, where the ideal cases are marked by diagonal dashed lines.

**Figure** **2** illustrates the retrieval of dimensions of elliptical holes from single‐shot images recorded experimentally with phase singularity illumination using superoscillatory light. E.g., Figure 2a shows the image recorded for an elliptical hole with width = 9λ/61 and length = 171λ/244, where the phase singularity to the left of the hotspot in the superoscillatory field illuminates the elliptical aperture. After training the network with images of 160 of the 200 elliptical holes and their dimensions, it has the ability to retrieve the widths and lengths of unknown elliptical holes from unseen images, as shown in Figure 2b,c. These two panels show the retrieved and true widths and lengths from the test dataset (20 elliptical apertures with different dimensions) where each data point is the average of the answers given by 50 trained networks, which ensures stability and reduces the impact of outliers. The diagonal dotted line denotes a perfect mapping of predictions to actual dimensions. We characterize the performance of such optical metrology in terms of the standard deviation σ between predictions and actual dimensions, $\sigma \;=\sqrt{\frac{\sum_{i=1}^{n}{\left(X_{i}-X_{T,i}\right)}^{2}}{n}}$ where *X_i_* denotes the average of 50 answers from the neural networks and *X*_T_ *_,i_* is the true value of width or length, and *n* refers to the number of measurements (*n* = 20). As our experimental errors (Figure 2b,c) are dominated by a statistical spread around the true dimensions, our measurements may be considered accurate and σ is, therefore, a measure of the random statistical error, which is of order 18 nm (λ/27) for the width and 36.6 nm (λ/13) for the length of the elliptical holes. This achieved nanometer scale statistical error indicates the potential of the technique for the metrology of sub‐wavelength scale objects. It should further be noted that such measurements are fast, single‐shot without any need for scanning or object reconstruction, and therefore the measurement time in such an optical metrology can be as short as the image exposure time of the camera. Here, this is ≈100 ms, and it can be reduced further by the use of a faster, low‐noise camera (e.g., cooled) and brighter illumination (e.g., increased laser power).

**Figure 3:**
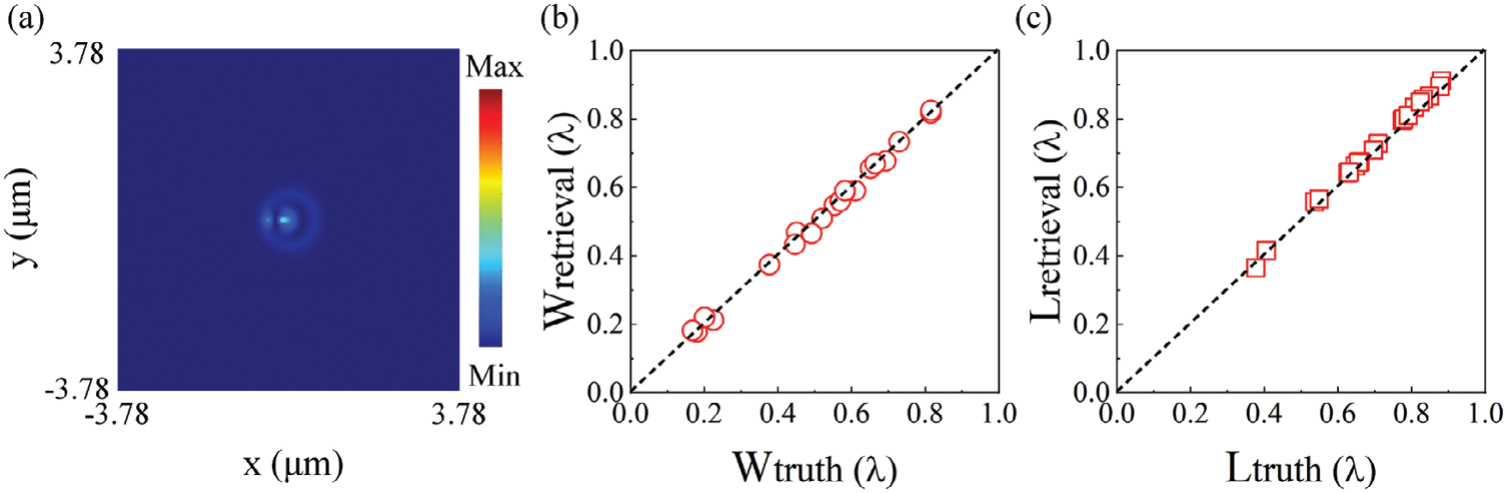
2D size retrieval of sub‐wavelength elliptical apertures based on simulated topological imaging. a) Simulated image of an elliptical hole (with the same size in Figure 2a) overlapping with a phase singularity of the superoscillatory light. b,c) Retrieved b) widths (circles) and c) lengths (squares) of elliptical apertures versus their actual sizes, where the ideal cases are marked by diagonal dashed lines and the neural network learns and predicts using simulated images.

To explore the potential lower statistical error in such optical metrology, we conduct numerical modeling under the same topological illumination condition but without any environmental instabilities, otherwise mirroring the experiments. We also acquire simulated images (e.g., **Figure** **3**a) of 200 elliptical apertures with the same sizes in the experiments and subsequently split them at random as training (160 images, 80%), validation (20 images, 10%), and testing (20 images, 10%) datasets for the same neural network processing. As shown in Figure 3b,c, the dimensions retrieved from the simulated images are accurate with significantly smaller statistical errors than those in the experiment. The standard deviations are 6 nm (λ/81) and 9.5 nm (λ/51) for width and length, respectively. This suggests that considerably reduced statistical errors approaching λ/81 may be achievable in such an optical metrology by minimizing fluctuations, e.g., associated with the alignment accuracy (Figure S3 and Note S3, Supporting Information), positional stability of the piezo stage (Figure S4 and Note S4, Supporting Information), mechanical stability of the optical setup and stability of the laser (wavelength, intensity, and polarization), which are affected by environmental temperature variation and vibrations.

We note that the width is retrieved with smaller statistical errors than the length of the elliptical holes in both experiments and simulations and suspect that this may be related to different phase (and amplitude) gradients of the incident field along these orthogonal directions.

**Figure 4:**
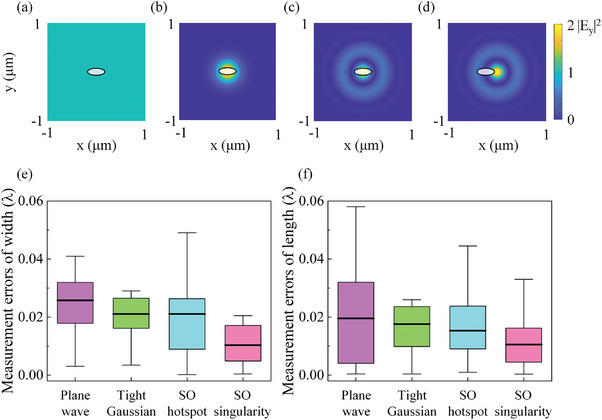
A comparison of measurement errors under different illumination conditions. a–d) Illumination diagrams of an elliptical hole under the plane wave, tightly‐focused Gaussian, superoscillatory (SO) hotspot, and SO singularity illumination, respectively. e,f) A comparison of measurement errors under such different illumination conditions according to simulations.

It is expected that the retrieval of the elliptical hole dimensions depends on the light field used for illumination. To investigate this, we also execute simulations and the neural network processing for plane wave, tightly‐focused Gaussian (with the full width at half maximum = 0.57 λ), and superoscillatory (SO) hotspot illumination of the same 200 elliptical holes, where all four types of incident light have the same wavelength and are shown in **Figure** **4**a–d. The measurement errors |*X_i_* − *X*_T,*i*_| for width and length of the elliptical holes (where *X_i_* denotes the average of 50 answers from the neural networks and *X*_T_ *_,i_* is the true value of width or length) are shown for all four illumination conditions in Figure 4e,f. The distribution of errors is represented by box plots, where the median lines provide insight into the typical measurement errors, which are 0.026 λ, 0.021 λ, 0.021 λ, and 0.010 λ for measured widths under plane wave, tightly‐focused Gaussian, SO hotspot, and SO singularity illuminations, as shown in Figure 4e. Their interquartile ranges (IQRs) are 0.014 λ, 0.010 λ, 0.017 λ, and 0.012 λ, respectively. The medians for the measured lengths are 0.020 λ, 0.018 λ, 0.015 λ, and 0.011 λ, with IQRs of 0.028 λ, 0.014 λ, 0.015 λ, and 0.012 λ under these four illumination conditions, respectively, as shown in Figure 4f. Both the median and the IQR indicate smaller measurement errors with superoscillatory singularity illumination, as compared to plane wave, tightly‐focused Gaussian, and superoscillatory hotspot illuminations. This indicates that superoscillatory singularity illumination with its giant phase gradients contributes to precise machine‐learning‐based retrieval of nano‐object dimensions from images.

While we have demonstrated the retrieval of two object parameters (width and length) of simple objects (elliptical holes), we anticipate that our metrology technique can be applied to more complex objects characterized by more object parameters, provided that the size of the training dataset is increased appropriately.

## 4 Conclusion

In summary, we have demonstrated both numerically and experimentally accurate 2D nanometrology with deep sub‐wavelength statistical errors which uses a neural network to retrieve the dimensions of a 2D sub‐wavelength object from its image recorded with topological illumination. This technique can characterize nano‐objects within milliseconds, i.e., within the time it takes to record a single‐shot image with a camera. Metrology accuracy and statistical errors surpassing the Abbe–Rayleigh diffraction limit in conventional microscopy dozens of times have been demonstrated here on a system allowing for the collection of physical training data for the neural network. More importantly, the technique does not use any fluorescent labeling, which avoids contamination and photo‐damage effects and broadens its potential in the engineering and biomedical fields, e.g., smart manufacture and quality control in the semiconductor industry and characterization of small structures in thin biomedical specimens.

## Conflict of Interest

The authors declare no conflict of interest.

## Author Contributions

Y. Wang and J. Y. Ou conceived the idea. Y. Wang and J. Y. Ou planned and carried out the experiments. Y. Wang planned and carried out the simulations. E. A. Chan constructed the neural network. C. Rendón‐Barraza prepared the sample. Y. Shen contributed to complex light field generation. E. Plum and J. Y. Ou supervised the work and contributed to the interpretation of the results. All authors provided critical feedback and helped shape the research, analysis, and manuscript. The authors are grateful to Prof. Nikolay I. Zheludev and Prof Kevin F. MacDonald for providing the experimental equipment and advice.

## Supporting information

Supporting Information

## Data Availability

The data that support the findings of this study are openly available in the University of Southampton ePrints research repository at https://doi.org/10.5258/SOTON/D3175.
