# Supplementary material for: 2D Super‐Resolution Metrology Based on Superoscillatory Light
Source: Adv Sci (Weinh). 2024 Aug 5;11(38):2404607. doi: 10.1002/advs.202404607 (PMC11481175; doi:10.1002/advs.202404607)
Supplement: Supplementary file 1 — Supporting Information [file ADVS-11-2404607-s001.pdf]

ADVANCED SCIENCE
Open Access

Supporting Information

for *Adv. Sci.*, DOI 10.1002/advs.202404607

2D Super-Resolution Metrology Based on Superoscillatory Light

Yu Wang, Eng Aik Chan, Carolina Rendón-Barraza, Yijie Shen, Eric Plum and Jun-Yu Ou**

Supporting Information

2D Super-Resolution Metrology Based on Superoscillatory Light

Yu Wang*[1], Eng Aik Chan[2], Carolina Rendón-Barraza[2], Yijie Shen[2], Eric Plum[1], Jun-Yu Ou*[3, 4]

1. Optoelectronics Research Centre & Centre for Photonic Metamaterials, University of Southampton, SO17 1BJ, United Kingdom
2. Centre for Disruptive Photonic Technologies, Nanyang Technological University, Singapore 637371, Singapore
3. School of Physics and Astronomy, University of Southampton, SO17 1BJ, United Kingdom
4. Institute for Life Sciences, University of Southampton, SO17 1BJ, United Kingdom
Wang.Yu@soton.ac.uk and bruce.ou@soton.ac.uk

S1. The experimental setup

The experimental setup of our 2D metrology system is displayed in Fig. S1. It consists of a dual microscope, a custom-built wavefront synthesizer system based on a pair of spatial light modulators, and a laser source [1]. The Gaussian beam from a laser with a wavelength of 488 nm is converted into a superoscillatory hotspot through a pair of spatial light modulators (SLMs), SLM-Amplitude and SLM-Phase, operating in amplitude and phase modulation modes, respectively. They convert the light into a balanced superposition of two circular prolate spheroidal wavefunctions (S_1 and S_2). This process creates a superoscillatory hotspot described by $E(r/\lambda) = 0.1206\ S_1(r/\lambda) + S_2(r/\lambda)$, where r is the radial distance from the beam axis [1, 2]. The resulting superoscillatory hotspot with a field of view of λ and a full width at half maximum of 0.42 λ is illustrated in Fig. 1 (b). The polarizers and the polarization controller are utilized to control and regulate the light to achieve linear y-polarization. The iris is used to remove unwanted diffraction orders from the SLMs. The lower objective lens (100× and NA=0.9) is utilized to focus the superoscillatory field to illuminate the object (i.e., an elliptical aperture, which is positioned by a piezoelectric stage), and the image of the object plane is collected by the camera through a second objective lens (100× and NA=0.9) in the transmission microscope.

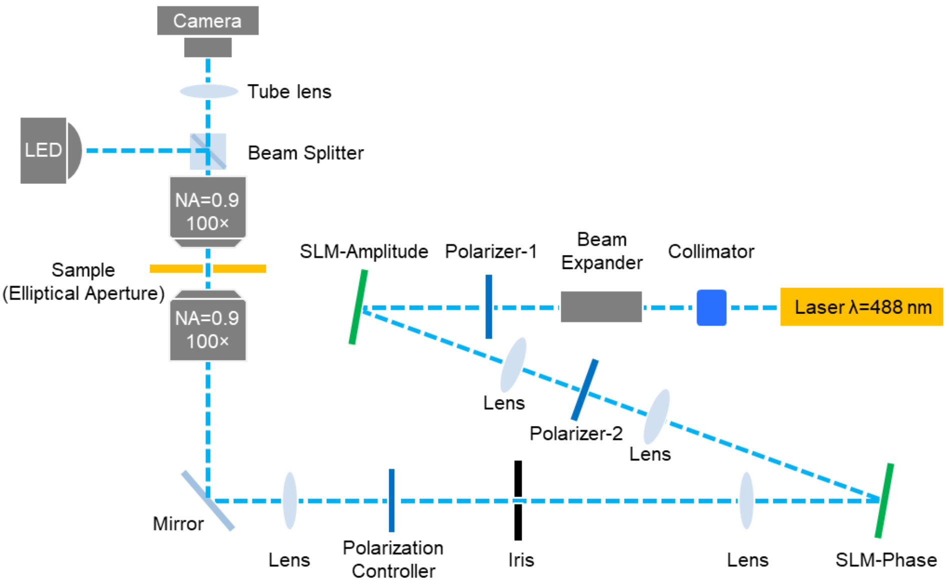

Fig. S1: Schematic diagram of the experimental setup.

S2. Focusing of superoscillatory field & alignment of elliptical hole with phase singularity

As illustrated in the experimental setup, a pair of spatial light modulators modifies the amplitude and the phase distribution of y-polarized incident light to achieve a superoscillatory hotspot. Then the hotspot is focused on the sample plane by the lower objective lens. We present here the intensity and phase distributions before [in Fig. S2 (a, b, c, and d)] and after [in Fig. S2 (e, f, g, and h)] the lower objective lens for orthogonal polarizations. In the generated superoscillatory field, there is a phase step area (phase singularity) located in the energy dark area between the hotspot and the halo (see ref. [3] for the definition of halo), as shown by two red dashed lines in Fig. S2 (c, d). When the superoscillatory hotspot is focused on the sample plane by the objective lens, a small x-component of electric field appears, but its intensity is negligible compared with the y-component, as illustrated in Fig. S2 (e, g), so that the total intensity pattern of the superoscillatory hotspot on the sample plane observed by the camera is dominated by the y-component. Although the intensity and phase distributions before and after the objective lens are not completely identical, the observed total intensity patterns and the region of the (first) phase singularity circling the hotspot are very similar.

In experimental measurements, the alignment of elliptical aperture with the phase singularity of the illuminating superoscillatory field is vital to achieve high measurement accuracy. We first find the energy dark area between the hotspot and the halo and mark the left phase singularity that will be used. Then we move the center of the elliptical aperture to align with the mark of the phase singularity, as illustrated in Fig. S2 (g, h).

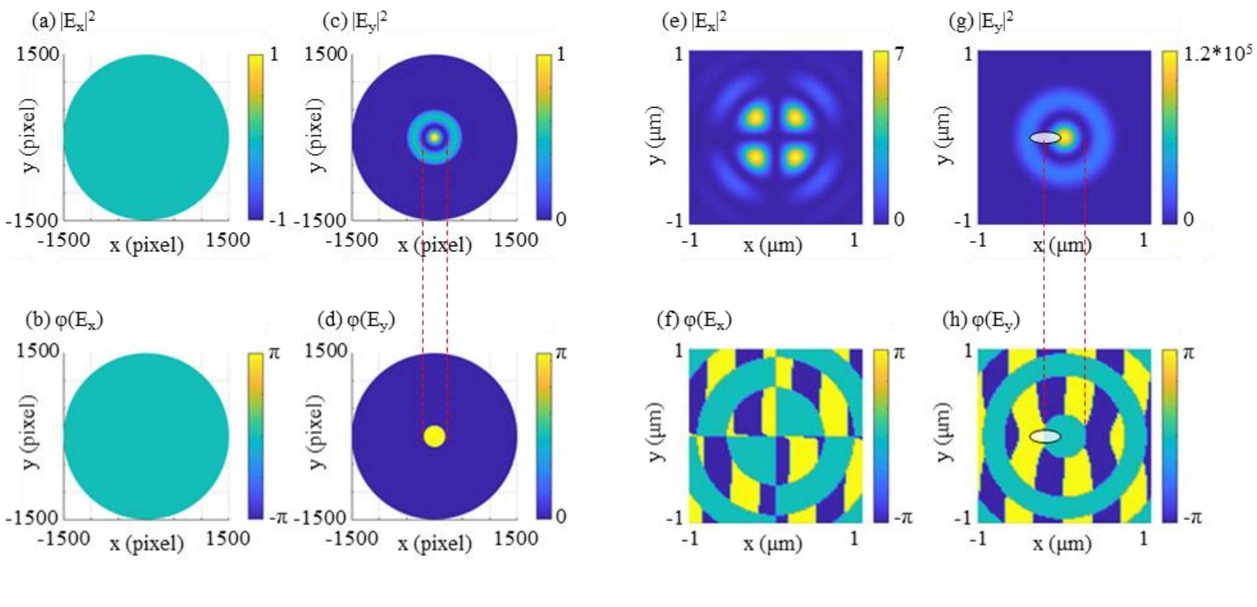

Fig. S2: The intensity (a, c, e, and g) and phase (b, d, f, and h) distributions of the superoscillatory field before (a, b, c, and d) and after (e, f, g, and h) the lower objective lens. The axis units of (a, b, c, and d) are in pixels on the SLMs.

S3. The influence of misalignment of phase singularity and elliptical hole on the random statistical error of the measurements

The alignment of the elliptical aperture with the phase singularity of the illuminating superoscillatory field is vital for such optical metrology to achieve small retrieval statistical errors in the experimental measurements. Here we investigate in simulations the impact of deviations between the positions of phase singularity and elliptical hole on the measurements, as shown in Fig. S3. The center of the elliptical hole randomly deviates from the phase singularity in the range of -3 nm and 3 nm in 3D, and other parameters are the same as that in Fig. 3. Compared with the simulated scenario where the phase singularity is precisely aligned with the elliptical aperture (Fig. 3), the measurement statistical errors deteriorate to 10 nm (λ/49) and 15.1 nm (λ/32) for width and length measurements respectively (Fig. S3), which highlights the significance of precise alignment in such optical metrology.

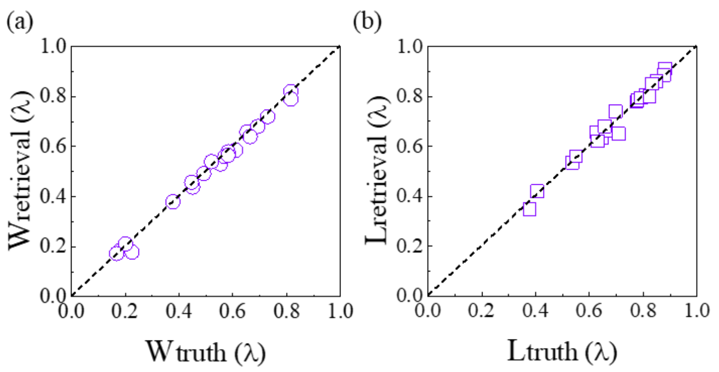

Fig. S3: 2D size retrieval of sub-wavelength elliptical apertures based on simulated topological imaging, with random 3D misalignment of up to ±3 nm between phase singularity and aperture. (a) Aperture width and (b) aperture length.

S4. The stability of the piezo stage

In the experiment, the sample with elliptical apertures is mounted on the piezo stage, which is used to position elliptical apertures at the phase singularity of the illuminating superoscillatory field for measurements. The instability of the piezo stage, influenced by vibrations from the environment, can result in an alignment error. Figure S4 characterizes the stability of the piezo stage by showing its positional error over time. We set the piezo stage at the (0,0,0) position, and record its actual position every 40 μs for 0.2 s. The position error ranges of the piezo stage are 3.5 nm, 2 nm, and 11.8 nm in x, y, and z directions respectively, which is one of the factors contributing to the experimental errors in such an optical metrology system.

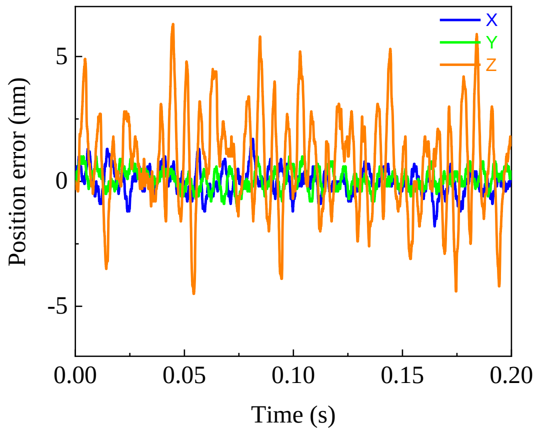

Fig. S4: Positional stability of the piezo stage.

References

[1] E. T. Rogers, S. Quraishe, K. S. Rogers, T. A. Newman, P. J. Smith, and N. I. Zheludev, "Far-field unlabeled super-resolution imaging with superoscillatory illumination," *Apl Photonics,* vol. 5, no. 6, p. 066107, 2020.

[2] K. S. Rogers, K. N. Bourdakos, G. H. Yuan, S. Mahajan, and E. T. Rogers, "Optimising superoscillatory spots for far-field super-resolution imaging," *Optics Express,* vol. 26, no. 7, pp. 8095-8112, 2018.

[3] N. I. Zheludev and G. Yuan, "Optical superoscillation technologies beyond the diffraction limit," *Nature Reviews Physics,* vol. 4, no. 1, pp. 16-32, 2022.
